# The Costs and Cost-Effectiveness of a School-Based Comprehensive Intervention Study on Childhood Obesity in China

**DOI:** 10.1371/journal.pone.0077971

**Published:** 2013-10-18

**Authors:** Liping Meng, Haiquan Xu, Ailing Liu, Joop van Raaij, Wanda Bemelmans, Xiaoqi Hu, Qian Zhang, Songming Du, Hongyun Fang, Jun Ma, Guifa Xu, Ying Li, Hongwei Guo, Lin Du, Guansheng Ma

**Affiliations:** 1 National Institute for Nutrition and Food Safety, Chinese Center for Disease Control and Prevention, Beijing, China; 2 National Institute for Public Health and the Environment, Bilthoven, The Netherlands; 3 Beijing University Health Science Center, Beijing, China; 4 Shandong University, Jinan, China; 5 Public Health College, Harbin Medical University, Harbin, China; 6 School of Public Health, Fudan University, Shanghai, China; 7 Guangzhou Center for Diseases Prevention and Control, Guangzhou, China; Groningen Research Institute of Pharmacy, Netherlands

## Abstract

**Background:**

The dramatic rise of overweight and obesity among Chinese children has greatly affected the social economic development. However, no information on the cost-effectiveness of interventions in China is available. The objective of this study is to evaluate the cost and the cost-effectiveness of a comprehensive intervention program for childhood obesity. We hypothesized the integrated intervention which combined nutrition education and physical activity (PA) is more cost-effective than the same intensity of single intervention.

**Methods:**

**And Findings**: A multi-center randomized controlled trial conducted in six large cities during 2009-2010. A total of 8301 primary school students were categorized into five groups and followed one academic year. Nutrition intervention, PA intervention and their shared common control group were located in Beijing. The combined intervention and its’ control group were located in other 5 cities. In nutrition education group, ‘nutrition and health classes’ were given 6 times for the students, 2 times for the parents and 4 times for the teachers and health workers. "Happy 10" was carried out twice per day in PA group. The comprehensive intervention was a combination of nutrition and PA interventions. BMI and BAZ increment was 0.65 kg/m^2^ (SE 0.09) and 0.01 (SE 0.11) in the combined intervention, respectively, significantly lower than that in its’ control group (0.82±0.09 for BMI, 0.10±0.11 for BAZ). No significant difference were found neither in BMI nor in BAZ change between the PA intervention and its’ control, which is the same case in the nutrition intervention. The single intervention has a relative lower intervention costs compared with the combined intervention. Labor costs in Guangzhou, Shanghai and Jinan was higher compared to other cities. The cost-effectiveness ratio was $120.3 for BMI and $249.3 for BAZ in combined intervention, respectively.

**Conclusions:**

The school-based integrated obesity intervention program was cost-effectiveness for children in urban China.

**Trial Registration:**

Chinese Clinical Trial Registry ChiCTR-PRC-09000402 URL:http://www.chictr.org/cn/

## Introduction

During the past couple of decades, China has experienced rapid socio-economic and nutritional transitions [1-4]. Along with these life style changes, the prevalence of overweight and obesity among Chinese children has more than tripled, from 1.7% in 1982 to 5.3% in 2002 for 7-12 years of age [5]. The dramatic rise of overweight among children has led policy makers to rank it as a critical public health threat for several reasons. Firstly, childhood obesity are more likely to persist into adulthood [6,7]. Secondly, obesity in adults is one of the main risk factors for some chronic diseases [8]. Finally, the obesity epidemic greatly affects the social economic development. The indirect effects of obesity and obesity-related dietary and physical activity patterns reached 3.4% of gross national product (GNP) in 2000 and was projected to reach 8.7% in 2025 [9].

 Schools have been identified as a key setting for public health strategies to prevent childhood obesity [10]. Research has shown that well-designed, well-implemented school obesity prevention programmes were effective in reduction of body mass index (BMI) and/or percent of body fat (PBF) [11]. However, some systematic reviews show that some short-term interventions (

< 12 month) focused on combining dietary and physical activity approaches did not significantly decrease BMI [12,13]. In China, a few studies indicated that school based comprehensive intervention combined with nutrition and physical activity programs were effective [14,15]. However, whether it would be successful when expanded to a larger scale (from more regions to national-wide) still remains unclear. 

As public health resources are limited, policy makers have to focus on how to set priorities among numerous public health issues. Therefore, interventions must not only be effective but also be cost-effective. Unfortunately, little information on the cost-effectiveness of different interventions is available in China. Under this background, this study was developed by National Institute for Nutrition and Food Safety (NINFS), Chinese Center for Disease Control and Prevention (China CDC), and funded by Ministry of Science and Technology of the People's Republic of China. The objective of this study is to evaluate the effects and the cost-effectiveness of a comprehensive intervention program for childhood obesity which combined nutrition education and physical activity interventions vs. control. We hypothesized the integrated intervention is both more effective in improving children’s BMI and also more cost-effective than the same intensity of single nutrition education intervention or physical activity intervention.

## Methods

### Study design

This study is a multi-center randomized controlled trial. Six centers included Beijing, Shanghai, Chongqing, Guangzhou, Jinan and Harbin were recruited. Two-step cluster sampling was used for subject selection. In the first step, 9 schools in Beijing were selected and assigned randomly to nutrition intervention (3 schools), physical activity (PA) intervention (3 schools) or control condition (3 schools). In other five cities, 6 schools in each city were selected randomly assigned to either combined with nutrition education and PA intervention (3 schools) or control condition (3 schools). Thus, there are a total of 15 schools in combined intervention and 15 schools in the control group in other 5 cities. In the second step, 2 classes from each grade in each school were chosen randomly.

No intervention was taken place in the control schools. 

### Sample Size Calculations

According to the study protocol, we calculated the sample size on the basis of cluster randomization trial design. “BMI changes” was used as the variable to calculate sample size. Based on the data from the 2002 China National Nutrition and Health Survey (CNNHS), a 0.7 kg/m^2^ of BMI reduction could gain 2 percent reduction of overweight and obesity prevalence. A school-based obesity intervention strategy which gain 2 percent obesity prevalence reduction was considered as an effective strategy by World Health Organization [16,17]. We assumed that the intervention would be effective if a difference of 0.7 kg/m^2^ of BMI changes between the intervention and control groups was detected. The intra-class correlation was assumed to be 0.05 and the power to detect the difference was set at 0.9. Statistical significance was set at 5% (two-sided). Thus the minimum number required would be 6 schools (3 for intervention and 3 for control) in each center with 250 students in each school. With consideration of loss of follow-up, the sample size of 9750 would be adequate. 

### Participants

The schools which meet the inclusion criteria (① non-boarding school; ② the students' overweight & obesity rate is over 10%; ③ school feeding, and more than 50% of the student eat lunch at school) were randomly chosen into the trial by a random number table. All of the students in the selected classes were enrolled in the trial, expect that: ① the student who suffer from serious illnesses, such as congenital heart disease, the body carried out fixation or joint replacement surgery, and so on, can not withstand severe physical activity and diet control, not to participate in this study; ② participated in the past one year or plan to participate in other similar intervention projects within one year. 

### Ethics Statement

This study was approved by the Ethical Review Committee of NINFS, China CDC. All participant students and their parents signed informed consent voluntarily. 

### Intervention

The program was implemented for 2 semesters during one academic year (May 2009 to May 2010). Three means of intervention were included in the present study: nutrition education, physical activity intervention and comprehensive intervention.

#### Nutrition education intervention

The nutrition education handbook [18] was developed by the Department of Student Nutrition, NINFS, China CDC. Carton pamphlets were distributed to each student in the intervention schools. Class on nutrition and health were given 6 times for the students, 2 times for the parents and 4 times for teachers and health workers. The menu for students of school lunch cafeteria was evaluated periodically and specific nutrition improvement was suggested accordingly. 

#### PA intervention

A classroom-based physical activity program for elementary students named “Happy 10” [19] was used in PA intervention. In each school day, the students were conducted “Happy 10” led by teachers to do a 10-minute segment moderate intensity, age- and space-appropriate exercises. The form of exercises was game, dance or rhythmic gymnastics. Students were also encouraged to develop more forms of exercises they like. Furthermore, education about physical activity was provided to students, parents, health workers and teachers. Each student attended the “Happy 10” 10 minutes for once, twice a day or 20 minutes for each time, once a day. 

#### Comprehensive intervention

The comprehensive intervention was a combination of nutrition and PA interventions. Detailed information on the interventions can be found in previous published article [20].

### Outcome measures

Measurements were collected at baseline as well as at the end of the intervention. Consistent assessment methods were used throughout the study. Height was measured to an accuracy of 0.1 cm with a freestanding stadiometer mounted on a rigid tripod. Fasting body weight was measured to the nearest 0.1 kg on a digital scale. BMI was calculated as weight in kilograms divided by the square of height in meter (kg/m^2^). Overweight was defined as BMI between the 85th and the 95th percentiles, whereas obesity was defined as BMI ≥ 95th percentile, using age- and sex-specific BMI cutoff points developed by the Working Group for Obesity in China (WGOC) [21]. 

#### Costs collection and calculation

The cost was collected and assessed from the social perspective. The project coordinator in each center was asked to recall all the costs related to the program. There are two components of the total costs in this paper. One is the 'Money costs' and the other one is the labor costs. Money costs means the direct currency investment during the program implementing. Labor costs was the transform of labor time investment. Three aspects data concerning costs were collected: a) Intervention costs in intervention schools; b) Evaluation cost for the pre- and post-intervention; and c) The development cost on structuring the program before intervention. 

 To estimate the intervention costs, we considered all costs associated with implementing the program during the intervention period, including expenses for materials, training, communication, transportation and accommodation, and monitoring. Material expenses included the cost of education material printing (Program Handbooks, nutrition and physical activity education book, pamphlet and foldout, dietary guideline manual), food pagoda model, “Happy 10” CD and poster. Training costs included meeting room, food, accommodation and training supplies as well as honorarium of instructors participating in the preprogram training. Communication costs included all the costs of communication meetings in which people shared the experiences and the limitations and would improve further in the future on the intervention during the intervention period. Transportation costs included traveling tickets for training and communication, the charge for taxi and compensation for transportation administrators. Monitoring costs included all the costs related to monitoring in each level, such as traveling and accommodation during the process of going to the intervention schools supervising, meetings for intervention and compensation of instructors participating in the monitoring**.**

To estimate the evaluation costs, we collected all the costs related to baseline and post-intervention survey implement and assessment, including questionnaire printing, instruments and tools for physical examination, expendables and allowance for blood samples taking, reagent and tests cost for biochemistry indices determination and related transportation cost. 

 Labor investment included the time been taken for preparing, implementing and evaluating the program. We collected the information on the salary of staff who implemented the program at each level and their working days for the program, based on which we calculated the labor cost. Subsidy for students and short-term personnel was also considered as a part of labor costs. Labor costs was also classified as intervention cost, development cost and evaluation cost. The labor costs for development included designing the program, developing the protocol used for intervention evaluation and implement, drafting the intervention materials, organizing start-up meeting and mobilizing the staff in collaboration centers as well as in schools. Labor costs for evaluation included time investment in training, implementing two times survey, data input computer and statistical analysis, reporting, blood samples collection, transport and biochemistry index tests. Labor costs for intervention included time investment in implementing the intervention in intervention schools, staff in collaboration centers monitoring, communication meeting with staff in NINFS, in collaboration centers and in intervention schools. 

 All the costs were collected on the population level because all the cash investment and labor time investment were based on the population level. Cost per capita in each group was calculated by total intervention cost divided by total participants in each group.

### Cost-effectiveness analysis

The cost-effectiveness analysis was performed based on the results by comparing the changes between post-intervention and baseline in control schools with those in intervention schools. The effectiveness of the intervention was measured as BMI, BMI z-score (BAZ) reduction and cases of overweight & obesity prevented compared with the control condition. Cases of overweight & obesity prevented was calculated by the reduction of overweight & obesity prevalence multiply the participant numbers in intervention groups. Cost per capita was calculated with the number of the children who participated the baseline survey as the denominator. For BMI and BAZ, Cost-effectiveness ratio (CER) was calculated by cost per capita divided by reduction. While for case of overweight & obesity prevented, CER was calculated by total intervention cost divided by the overweight & obesity cases prevented. 

### Statistical methods

Results of the continuous variables were expressed as mean and standard error. Calculation and comparison of the means and the changes of continuous variables among intervention and control groups were used covariance analysis with General Linear Model (GLM) adjusted for age, gender, daily energy intake and leisure time physical activity per day (for comparing changes, the baseline index was as a additional adjusted variable). For comparison of the means between post-intervention with baseline within each group a paired t test was used. Overweight & obesity prevalence and its’ OR were compared using Generalized Linear Mixed Model (GLMM). Procedure GLMM was used to achieve it with center as random variable. The fixed variables included sex, age, baseline BMI and intervention types. 

Statistical significance was set at *P*<0.05. SAS package version 9.1 (SAS Institute Inc, Cary, NC) was used for analyses. 

## Results

A total of 9750 primary school students (aged 6-13 years) participated in the survey at baseline. Totally, 114 of the 5250 participants in intervention group and 309 of the 4500 participants in control group declined to participate. A total of 123 students were lost to follow-up because of school transfer or moving and 156 students accepted discontinued intervention because of illness and dropout in intervention group. According to the study protocol, the trial ended in May, 2010, after one academic year’s intervention. As a result, a total of 8301 students followed full term (mean duration was 8.9±0.1 months) intervention with logical general information and anthropometric index. Totally, 615 and 590 students completed the nutrition intervention and PA intervention in Beijing, respectively while 3356 participants completed the combined intervention in other 5 centers. 

 The percentage of males in the control group in Beijing was significantly higher than that in the two single intervention groups, however, no significant differences were found between the combined intervention and the control group in the other 5 centers. The distribution of age among control and intervention groups in Beijing was consistent, which was also seen between the control and the combined intervention group in other 5 centers. No significant differences were found in income, leisure time activity, sedentary activity and energy intake between combined intervention group and its’ control group at baseline. However, children in combined intervention were less active and lower energy intake compared with its’ control counterparts (Table 1).

**Table 1:** Characteristics of the subjects at baseline by group.

	Beijing							Other 5 centers			
	Control		Nutrition intervention		PA intervention			Control		Nutrition & PA intervention	
	N	%	N	%	N	%		N	%	N	%
**Total**	460	100.0	615	100	590	100		3280	100	3356	100
**Sex**											
Male	266	57.8^a^	300	48.8^b^	302	51.2^b^		1644	50.1	1695	50.5
Female	194	42.4	315	51.2	288	48.8		1636	49.9	1661	49.5
**Age**											
6-9.9	314	68.3	427	69.4	420	71.2		2357	71.9	2381	70.9
10-13.9	146	31.7	188	30.6	170	28.8		923	28.1	975	29.1
	%							%			
Income capita per month (yuan, RMB)											
<750	14.2		10.9		16.7			11.8		10.4	
750-2500	67.1		66.8		64.2			60.5		59.2	
≥2501	18.7		22.4		19.1			27.7		30.5	
*P^c^*			0.34		0.70					0.08	
Leisure time activity per day											
<1h	24.0		17.6		15.8			40.5		39.5	
1~1.9h	15.1		28.2		33.1			21.6		21.4	
2~2.9h	19.6		23.2		22.0			14.3		17.4	
≥3h	41.3		30.9		29.0			23.6		21.7	
*P^c^*			0.02		<0.001					0.21	
Sedentary activity per day											
<1h	62.2		61.8		63.9			51.6		52.8	
1~1.9h	33.8		33.5		33.7			38.2		37.2	
≥2h	4.0		4.7		2.3			10.1		10.1	
*P^c^*			0.86		0.20					0.44	
Average energy intake per day (Kcal) , Mean (std)											
	1077.6(428.4)^a^		1036.4(403.1) ^a^		929.4(388.9) ^b^			1467.2(713.8)		1356.1(569.1)^*^	

^a^ ^b^: Percentage shared the different letter means significant difference at baseline among groups in Beijing, p<0.05. ^*^ Significant difference (p<0.05) between control and Nutrition & PA intervention group. ^c^ statistical analysis and compare between intervention group with it’s control group. No superscript means no significant difference among groups.

Table 2 showed anthropometric characteristics at baseline, after intervention and the changes between post-intervention and baseline. No significant differences were found in height, weight and BMI between the nutrition intervention group and its’ control group at baseline. Height, weight and BMI in each group in Beijing were significant higher in post intervention than those of at baseline. In the combined intervention group in the other 5 centers, height increasing was significantly more (6.82 cm vs. 6.59 cm) while weight, BMI and BAZ increasing were significantly less than those in its’ control group (4.75 kg vs. 4.95 kg, 0.65 kg/m^2^ vs. 0.84 kg/m^2^, 0.12 vs 0.20, respectively). No significant differences were found in BMI change (after and before intervention) between the PA intervention group and its’ control group in Beijing, the same case is for nutrition intervention group in Beijing. No significant differences were found in the changes of the overweight and obesity prevalence among nutrition intervention group, PA intervention group and their control group. However, the increment of the overweight and obesity prevalence in combined intervention group in other 5 centers was 87% less than that in control group, with a borderline significant difference (OR and 95% CI: 0.9 (0.7, 1.0)), see details in Table 2. 

**Table 2:** Anthropometric characteristics and obesity prevalence at baseline, after intervention and changes in different groups**^**.

			Beijing				Other centers
		Control	Nutrition intervention	PA intervention		Control	Nutrition & PA intervention
**Height (cm)**							
N		460	615	590		3280	3356
Baseline		135.76±.00^a^	136.75±0.98^a^	136.20±0.97^a^		137.64±0.45	137.80±9.0.46
Post-intervention		141.36±1.05^*^	142.65±1.03^*^	141.91±1.02^*^		144.36±0.49^*^	144.77±0.49^*^
Change		5.60±0.27^c^	5.90±0.26^d^	5.71±0.26^cd^		6.59±0.14	6.82±0.14^##^
**Weight (kg)**							
N		460	615	590		3280	3356
Baseline		30.66±1.11^a^	31.57±1.09^ab^	31.90±1.08^b^		32.81±0.52	32.76±0.53
Post-intervention		34.77±1.33^*^	35.95±1.31^*^	36.22±1.30^*^		37.86±0.63^*^	37.62±0.64^*^
Change		4.10±0.38^c^	4.38±0.37^c^	4.33±0.37^c^		4.95±0.19	4.75±0.20^#^
**BMI (kg/m^2^)**							
Baseline		16.42±0.43^a^	16.66±0.42^ab^	16.95±0.42^b^		17.07±0.20	17.01±0.20
Post-intervention		17.14±0.48^*^	17.40±0.67^*^	17.72±0.46^*^		17.91±0.23^*^	17.68±0.23^*^
Change		0.72±0.15^c^	0.74±0.15^c^	0.76±0.15^c^		0.84±0.09	0.65±0.09^##^
BAZ (BMI Z-score)							
Baseline		-0.17±0.19^a^	-0.05±0.18^ab^	0.06±0.18^b^		0.05±0.09	0.03±0.09
Post-intervention		0.07±0.19^*^	0.20±0.19^*^	0.31±0.18^*^		0.25±0.10^*^	0.15±0.11^*^
Change		0.25±0.07^c^	0.25±0.06^c^	0.26±0.06^c^		0.20±0.04	0.12±0.05^##^
**Overweight and Obesity, n (%)**							
Baseline	51 (11.1)		88 (14.3)	95 (16.1)		746 (22.7)	792 (23.6)
Post-intervention	81 (17.6)		122 (19.8)	129 (21.9)		795 (24.2)	798 (23.8)
*Changes*	*30 (6.5)*		*34(5.5)*	*34(5.8)*		*49 (1.5)*	*6 (0.2)*
*P*			*0.24*	*0.17*			*0.06*
*OR* (*95% CI*) ^†^	*1.0*		*0.8(0.0, 18.8)*	*0.94 (0.0, 20.8)*		*1.0*	*0.9(0.7, 1.0)*

^^^ Continuous variables were expressed as mean±standard error.

^a, b^: Means shared the different letter means significant difference at baseline among groups in Beijing, p<0.05.

^c, d^ : Means shared the different letter means significant difference of changes (post-intervention vs. baseline) among groups in Beijing, p<0.05.

^*^ Comparison the mean between post-intervention and baseline in each group, p< 0.05.

^#^ Comparison means between combined intervention group and control group at baseline as well as for changes (post-intervention vs. baseline), ^#^ p<0.05; ^##^ p<0.01.

^†^ OR and 95% CI for overweight & obesity prevalence using generalized linear mixed model, no significantly difference of OR between nutrition or PA individual intervention group with their control group, but a borderline difference between combined intervention group with its’ control group (p=0.06).

The costs of development and evaluation of the program was shown in Table 3. The total development costs in combined intervention group was RMB 26619 ($3915) for 5 collaboration centers. The development costs in nutrition intervention and PA intervention group was the same amount, RMB 4769 ($701). The development costs in each control group was zero. The total evaluation costs was RMB 173513 ($25517), RMB 141873 ($20864) and RMB 978614 ($143914) in nutrition intervention, PA intervention and combined intervention group, respectively. 

**Table 3:** The costs of development and evaluation of the program (RMB (US dollars)^*^).

Categories	Beijing				Other centers	
	Control	Nutrition intervention	PA intervention		Control	Nutrition & PA intervention
**Development Costs**						
*Money costs*	0 (0)	1817 (267)	1817 (267)		0 (0)	2425 (357)
*Labor costs*	0 (0)	2952 (434)	2952 (434)		0 (0)	24194 (3558 )
*Total*	0 (0)	4769(701)	4769(701)		0 (0)	26619(3915)
**Evaluation Costs**						
*Money costs subtota*l	137510 (20222)	153010 (22501)	108310 (15928)		745904 (109692)	738978 (108673)
Materials	92592 (13616)	103792 (15264)	59592 (8764)		537372 (79025)	539311 (79310)
Training	2546 (374)	729 (107)	729 (107)		22718 (3341)	14656 (2155)
Personnel allowance	12489 (1837)	15589 (2293)	15089 (2219)		119693 (17602)	119429 (17563)
Transport and accommodation	31800 (4676)	33000 (4853)	33000 (4853)		47057 (6920)	39217 (5767)
Collaborate fee	0 (0)	0 (0)	0 (0)		29250 (4301)	34126 (5019)
*Labor costs*	25691 (3778)	20503 (3015)	33563 (4936)		22801 (3353)	239636 (35241)
*Total evaluation costs*	163201 (24000)	173513 (25517)	141873 (20864)		768705 (113045)	978614 (143914)

* US dollars was calculated by Jan, 2010 exchange rate (6.8).

The intervention costs was shown in Table 4. The intervention costs per child in combined intervention group was RMB182.4 ($26.8), which was 2.4 times higher than that in the nutrition intervention (RMB52.8, $7.8) or in the PA intervention (RMB52.3, $7.7). The money costs per child in combined intervention was 25.5 RMB ($3.8), which is lower then that in the individual intervention group ($4.7 for nutrition intervention and $4.4 for PA intervention). However, the labor costs per child was much higher in combined intervention ($23.0) compared with individual intervention and accounted for 86% of total costs. Guangzhou, Shanghai and Jinan in combined intervention group had the highest labor costs. 

**Table 4:** Cost of intervention in the intervention schools (RMB (US dollars)).

	Nutrition intervention	PA intervention	Nutrition & PA intervention					
			Jinan	Guangzhou	Shanghai	Harbin	Chongqing	Subtotal
***Money Costs***								
Materials	4414 (649.1)	2593 (381.3)	6544 (962.4)	6372 (937.1)	5774 (849.1)	5959 (876.3)	4204 (618.2)	28853 (4243.1)
Training	3074 (452.1)	3074 (452.1)	3426 (503.8)	6914 (1016.8)	5427 (798.1 )	4351 (639.9)	5410 (795.6)	25528 (3754.1)
Communication	1453 (213.7)	1453 (213.7)	3309 (486.6)	5350 (786.8)	4480 (658.8)	3850 (566.2)	4470 (657.4)	21459 (3155.7)
Transportation and accommodation	7800 (1147.1)	7800 (1147.1)	1700 (250.0)	1080 (158.8)	5220 (767.6)	1300 (191.2)	3900 (573.5)	13200 (1941.2)
Monitoring	8300 (1220.6)	8300 (1220.6)	5100 (750.0)	800 (117.6)	1440 (211.8)	500 (73.5)	2000 (294.1)	9840 (1447.1)
*Subtotal*	25041 (3682.5)	23220 (3414.7)	20079 (2952.8)	20516 (3017.1)	22341 (3285.4)	15960 (2347.1)	19984 (2938.8)	98880 (14541.2)
***Labor costs***								
School Intervention	10088 (1483.5)	10661 (1567.8)	95756 (14081.8)	87060 (12802.9)	235721 (34664.9)	66932 (9842.9)	4542 (667.9)	490011 (72060.4)
Center monitoring	5964 (877.1)	5964 (877.1)	27108 (3986.5)	15108 (2221.8)	41335 (6078.7)	8175 (1202.2)	23904 (3515.3)	115630 (17004.4)
National Communication§	638 (93.8)	638 (93.8)	638 (93.8)	638 (93.8)	638 (93.8)	638 (93.8)	638 (93.8)	3190 (469.1)
Subtotal	16690 (2454.4)	17263 (2538.7)	123502 (18162.1)	102806 (15118.5)	277694 (40837.4)	75745 (11139.0)	29084 (4277.1)	608831 (89534.0)
***Total costs||***	41731 (6136.9)	40483 (5953.4)	143581 (21114.9)	123322 (18135.6)	300035 (44122.8)	91705 (13486.0)	49068 (7215.9)	707711 (104075.1 )
***N***	790 (116.2)	774 (113.8)	693 (101.9)	1008 (148.2)	749 (110.1)	722 (106.2)	707 (104.0 )	3879 (570.4)
***Money costs per capita***	31.7 (4.7)	30.0 (4.4)	29.0 (4.3)	20.4 (3.0)	29.8 (4.4)	22.1 (3.3)	28.3 (4.2)	25,5 (3.8)
***Total costs per capita***	52,8 (7.8)	52,3 (7.7)	207,2 (30.5)	122,3 (18.0)	400,6 (58.9)	127,0 (18.7)	69,4 (10.2)	182,4 (26.8)

§ National communication costs in national lever was divided into each center in each intervention group.

|| Total costs means the sum of money costs and labor costs.

The cost-effectiveness results by center were shown in Table 5. Chongqing (one of the centers in combined intervention) as well as Beijing where the two individual interventions implanted were excluded in cost-effective analysis because that no consistent significant effect was found in these cities. We calculated the cost-effectiveness ratio separately in four cities (Jinan, Guangzhou, Shanghai and Harbin) in the combined intervention group, compared with controls, there was a 0.27 kg/m^2^, 0.29 kg/m^2^, 0.17 kg/m^2^ and 0.61 kg/m^2^ reduction of BMI attributing to the integrative intervention in Jinan, Guangzhou, Shanghai and Harbin, respectively. The cost for achieving 1 kg/m^2^ BMI reduction, CER was $113.0, $62.1, $346.5 and $30.7, respectively, in Jinan, Guangzhou, Shanghai and Harbin, The cost for avoided one overweight and obesity case was $1308.9.

**Table 5:** Cost-effectiveness ratio (CER) by city in the nutrition & PA combined intervention group.

Index	Jinan		Guangzhou		Shanghai		Harbin		Total^d^	
	Effect^a^	CER^b^	Effect	CER	Effect	CER	Effect	CER	Effect	CER
BMI (kg/m^2^)	0.27	113.0	0.29	62.1	0.17	346.5	0.61	30.7	0.29	120.3
BAZ	0.09	338.9	0.15	120.0	0.09	654.4	0.29	64.5	0.14	249.3
One case of O & B ^c^ prevented	21	1005.5	19	954.5	22	2005.6	12	1123.8	74	1308.9

^a^ For BMI, BAZ and overweight & obesity prevalence, the ‘effect’ means BMI, BAZ and overweight & obesity prevalence reduction (post intervention vs before intervention) in intervention group compared with that of in the control group, respectively. ^b^ ALL CER was presented in US dollars. ^c^ O & B means overweight & obesity. ^d^ Total’ means the average effect of four intervention centers (Jinan, Guangzhou, Harbin, Shanghai), Chongqing was excluded here because the intervention in this city was not effective (p>0.05).

## Discussion

### The costs of the intervention

The intervention costs in this study were lower compared with other similar intervention studies [10,22]. A study of a 3-year, after-school program to prevent obesity among elementary school students [10] reported the net intervention costs per capita was $317 and the achieved effectiveness was a 0.7% PBF reduction. Teaching materials both for PA activity and for nutrition education were simple and inexpensive in this study. Participates in the same class can share one set of material such as “Happy 10” video, tracking posters, and stickers, which was one of the measures to reduce costs. Furthermore, the space for taking PA activity intervention was in classroom or in the playground in school, which is costless. 

The money intervention costs per capita in the combined intervention group was relatively low in this study. However, the labor costs account for 86% of the total costs in the combined intervention group and the proportion was much higher compared to the single one. This may be due to the difference distance from the site national project office located to the intervention sites. As we known, the two single intervention groups was performed in Beijing, where the national working group located. So the traveling cost and time investment for training and supervision was saved largely. Meanwhile, the combined intervention was implemented in five centers separately. The organization, management and supervision were more difficult and needs more labor investment. There was also a variety of labor costs among the five combined intervention cities and the nonidentity of the salary income for the instructors and staff in collaboration centers maybe one of the reasons. For example, based on the questionnaire survey of labor cost, staff in Shanghai and Guangzhou subcenters have higher salary than those in other centers. Data from Statistical Bulletin for National Economic and Social Development showed that annual per capita disposable income in 2010 was RMB 34345 in Shanghai and RMB 34328 in Guangzhou, which was the two highest income levels in China.

### The Effectiveness of the Intervention

Up to now, there are not generalizable conclusion about the effects of child obesity interventions [11-13]. There was a borderline significant difference in change of the overweight & obesity prevalence (OR and 95% CI: 0.8(0.7, 1.0)) between the combined intervention and the control group. The increment in BMI was significantly lower in the combined intervention than that in the control group. It indicates that the combined intervention improves in obesity prevention. More effective function of combined intervention in obesity prevention was found in long-term intervention studies [14,23]. In the both two 3-year nutrition education & PA combined intervention studies, one [24] found a significant 1.1 kg/m^2^ reduction of BMI and another [14] found a significant 1.8 kg/m^2^ reduction of BMI. However, inconsistent results were also presented in other studies [24,25]. One 2-year combined intervention found no impact on obesity [25], in which the author suggested that compensation in both energy intake and physical activity outside of school may be responsible for the lack of difference between the intervention group and the control group. 

However, there was no effect found in BMI reduction in single intervention (PA or nutrition). Similar results were also found in other studies [26,27]. One possibility of this was that the “dose” of physical activity achieved in these studies was insufficient to improve BMI. A short term (10 months) but high intensity physical activity intervention was reported effectiveness and the reason for success, brought forward by the author, is partly the exercise ‘dose’ The average daily energy cost of the program is more than twice the magnitude of the proposed energy surplus associated with childhood obesity [28]. As similar with PA intervention, low intensity nutrition education intervention was also proved no effect neither in this study nor in others [29]. 

### The cost-effectiveness of the intervention

Most reports about the cost-effectiveness analysis of school-based child obesity intervention usually were based on per QALY (quality-adjusted life year) or DALY (disability-adjusted life-year) saved and studies based on BMI improvement was limited. We can’t convert BMI reduction into more meaningful measures such as QALYs or DALYs saved in this study due to the following reasons: 1) The information of the life quality of the subjects was in absence in this study and the quality weight was not available; 2) The effects in this study was not in strong evidence. Though there was a significant BMI reduction in combined intervention group, the reduction of overweight and obesity prevalence was not in statistical significance. Furthermore, the individual intervention group was not shown a significant effect compared with control group. 

The cost-effectiveness analysis results showed that CER for BMI in combined intervention was $120.3, which was much lower than that in one study implemented in Australia. In that study, the cost for achieving 1 kg/m^2^ BMI reduction was AUD 11236 after 12 weeks consultation intervention targeting change in nutrition, physical activity, and sedentary behavior based on family [30]. One score of BAZ reduction cost $249.3 and this was markedly lower than that in a family-based 12-month treatment for children obesity in Canada [31]. There was a large variation of the CER among combined intervention cities. Achieving one unit of BMI or BAZ reduction or one case of overweight & obesity prevented was quite expensive in Shanghai with the reason of high intervention cost and low effects. On the contrary to the high income in Shanghai, the annual per capita disposable income was the lowest (RMB 16292) in Harbin. As a result of low intervention cost ($18.7 per capita) and relative high effects, Harbin had the lowest CER for BMI and BAZ. 

 This study has several limitations. Firstly, the long-term effects and cost-effectiveness of the intervention can not be assessed. Secondly, although the intervention implemented for 2 semesters during one academic year, the actual implemented duration is 8.9 months because it was interrupted by the two regular holidays (one month summer holiday and two months winter holiday), which would reduce the expectable effects. Thirdly, the combined intervention was not implemented in the same center with the individual intervention. Although all the involved centers are located in large city, there are still differences in economical level, salary income and expenditure level among them and this make it less comparable in the cost (especially for the part of labor cost) and the cost-effectiveness among intervention groups. Finally, the sample size in single intervention group in Beijing is small and is not sufficient enough to detect the difference of changes. 

This study also had important strengths. Firstly, the program was standardized through the development (training of the instructors), intervention (uniform intervention materials) and evaluation (uniform measures and instruments for outcomes). Secondly, physical activity in style of “Happy 10” was a recreational and non-competitive. It was well acceptable and can avoid the danger of hurt during implementing. Thirdly, the parents, instructors, health workers, school canteen managers, operators were fully mobilized both in the nutrition education intervention and in the PA intervention, which played an important role on obesity prevention for students. Fourth, follow-up rates were high (85%) and similar rates among children in the control schools. This may be because parents knew in advance that biochemical results would be given shortly after the health examination at the end of the project, together with appropriate advice if any parameter was abnormal. 

A good design and full implementation are the keys to accomplish an intervention program. The methodology of an intervention program, such as duration, intensity, and the criteria of control selection, needs to be further studied. Evaluation the background level of the children’s physical activity and nutrition knowledge in both control schools and intervention schools are necessary. Future study needs to be conducted to identify whether the effects and the cost-effectiveness is sustainable in a long term, and a suitable model, such as QALYs, should be used to assess the cost-effectiveness for a long-term. 

In conclusion, the school-based integrated intervention was cost-effectiveness to improve BMI in school children and had a potential effect on childhood obesity prevention in urban China. However, the long-term effects and cost-effectiveness needs to be evaluated in the further study.
